# Self-Renewal and Pluripotency Acquired through Somatic Reprogramming to Human Cancer Stem Cells

**DOI:** 10.1371/journal.pone.0048699

**Published:** 2012-11-08

**Authors:** Shogo Nagata, Kunio Hirano, Michele Kanemori, Liang-Tso Sun, Takashi Tada

**Affiliations:** Department of Stem Cell Engineering, Institute for Frontier Medical Sciences, Kyoto University, Kyoto, Japan; University of Melbourne, Australia

## Abstract

Human induced pluripotent stem cells (iPSCs) are reprogrammed by transient expression of transcription factors in somatic cells. Approximately 1% of somatic cells can be reprogrammed into iPSCs, while the remaining somatic cells are differentially reprogrammed. Here, we established induced pluripotent cancer stem-like cells (iCSCs) as self-renewing pluripotent cell clones. Stable iCSC lines were established from unstable induced epithelial stem cell (iESC) lines through re-plating followed by embryoid body formation and serial transplantation. iCSCs shared the expression of pluripotent marker genes with iPSCs, except for *REX1* and *LIN28*, while exhibited the expression of somatic marker genes *EMP1* and *PPARγ*. iESCs and iCSCs could generate teratomas with high efficiency by implantation into immunodeficient mice. The second iCSCs isolated from dissociated cells of teratoma from the first iCSCs were stably maintained, showing a gene expression profile similar to the first iCSCs. In the first and second iCSCs, transgene-derived *Oct4*, *Sox2*, *Klf4*, and *c-Myc* were expressed. Comparative global gene expression analyses demonstrated that the first iCSCs were similar to iESCs, and clearly different from human iPSCs and somatic cells. In iCSCs, gene expression kinetics of the core pluripotency factor and the Myc-related factor were pluripotent type, whereas the polycomb complex factor was somatic type. These findings indicate that pluripotent tumorigenicity can be conferred on somatic cells through up-regulation of the core pluripotency and Myc-related factors, prior to establishment of the iPSC molecular network by full reprogramming through down-regulation of the polycomb complex factor.

## Introduction

Cancer stem cells (CSCs), which are subpopulations of tumor cells, function in maintaining cancers through initiation and propagation of perpetuating tumor growth [1]. CSCs are thought to be key targets of cancer therapies, but the details of their genetic and epigenetic signatures are unclear. As a gold standard to define CSC properties, a serial transplantation assay based on the ability to self-renew and generate tumors has been widely used [2]. A crucial event in initiating cancers is activation of the self-renewal machinery, which is normally limited to stem cells. Therefore, it is likely that CSCs share several gene expression signatures detected in pluripotent stem cells. In fact, pluripotent marker genes, *Oct4* and *Nanog*, are expressed in some cancers [3], [4], and oncogene *Myc* is involved in the generation of many cancers [5].

Forced expression of a combination of transcriptional factors, Oct4, Sox2, Klf4, and c-Myc (OSKM), can promote direct reprogramming of human and mouse somatic cells into induced pluripotent stem cells (iPSCs) [6], [7]. In direct reprogramming, *Oct4* and *Sox2*, which are core pluripotency factors, function as regulators of developmental and transcription-associated processes [8], *Myc* targets genes predominantly involved in cellular metabolism, cell cycle, and protein synthesis pathways [9]. Furthermore, *Myc* functions to increase efficiency by regulating the p53 pathway [10]. This evidence indicates that common pathways could be used both in the acquisition of pluripotency and tumorigenesis. In humans, CSC-like cells were transformed from primary skin fibroblasts by the stable expression of hTERT, H-RasV12, and SV40 LT and ST antigens [11]. In mice, CSCs were generated from mouse induced pluripotent stem cells (iPSCs) by culture with a conditioned medium of cancer cell lines, which was a mimic of the carcinoma microenvironment [12]. Thus, global change of the transcription signature through direct reprogramming or alternative culture conditions could promote the transformation to CSCs. In this context, it is possible that forced expression of OSKM in somatic cells induces direct reprogramming into CSCs. To address the molecular mechanisms involved in embryonic stem (ES) cells and CSCs, three functionally different gene sets, called the Core (core pluripotency factors), PRC (polycomb repressive complex factors), and Myc (Myc-related factors) modules, proposed recently were used for comparative analyses of global gene activity between different types of cells [9].

Here, in order to address questions of whether human induced cancer stem-like cells (iCSCs) can be generated by somatic reprogramming through conventional OSKM viral induction, and how human iCSCs, but not iPSCs, are generated, first we isolated iCSCs from cell populations, which acquired the ability to self-renew after forced expression of exogenous OSKM in human somatic fibroblasts TIG1. iCSCs have the property of pluripotency as verified by teratoma formation through serial transplantation to immunodeficient mice. Notably, the gene expression signature demonstrated that iCSCs persist certain somatic cell memory even after up-regulation of pluripotent marker genes through reprogramming. Our findings revealed that up-regulation of gene sets for the Core and Myc modules is sufficient to confer the properties of self-renewal and pluripotency, and sequential down-regulation of gene sets for the PRC module is required to install the proper iPSC signature on somatic cells. These findings demonstrate that iCSCs and iPSCs share a reprogramming pathway from somatic nuclei into pluripotent and self-renewable nuclei, and then diverge to iCSCs or iPSCs.

## Materials and Methods

### Ethics statement

Experiments with mice were performed according to the institutional guideline of Kyoto University, Japan. Our animal experiments (W-3-6) are reviewed and permitted by the animal research committee of Kyoto University, Japan.

### Cell culture

Human fetal lung fibroblasts (TIG1) provided by the JCRB Cell Bank were cultured in Dulbecco's modified Eagle's medium (DMEM) (Sigma-Aldrich, USA) containing 10% FBS, and were infected with Oct4, Sox2, Klf4 and c-Myc retroviruses. At day 4 after infection, the cells were reseeded into a 10 cm culture dish on feeder cells. At day 5 after infection, culture medium was changed to iPSC medium (DMEM/Nutrient Mixture F-12 Ham (DMEM/F12) (Sigma-Aldrich) supplemented with 20% of knockout serum replacement (Invitrogen, USA), 10 ng/ml bFGF (Peprotech, USA), L-glutamine, and non-essential amino acids and 2-mercaptoethanol). Colonies, which could self-renew and expand, were picked up and reseeded onto feeder cells around day 30. To isolate human iPSCs and induced epithelial stem cells (iESCs), each colony was picked up and reseeded into Matrigel-coated dishes with mouse embryonic fibroblast (MEF)-conditioned iPSC medium.

For embryoid body (EB) formation, small iESC aggregates were formed by overnight hanging drop culture in MEF-conditioned iPSC medium. Aggregates were grown in bacterial culture dishes for 5–7 days, and then cultured in gelatin-coated dish with DMEM containing 10% FBS.

For tumor-derived cell culture, tumors were cut into small pieces, dissociated with collagenase, and plated on gelatin-coated dishes with 10% FBS containing DMEM. Established iCSC lines were maintained in gelatin-coated dish with DMEM/F12 (Wako, Japan) supplemented with 15% FBS, 1000 U/ml LIF (Chemicon, USA), L-glutamine, penicillin-streptomycin, sodium bicarbonate, sodium pyruvate, and 2-mercaptoethanol through passage using 0.25% trypsin/EDTA.

### RT-PCR

For RT-PCR analyses, total RNA of cultured cells was extracted with TRIzol reagent (Invitrogen). cDNA was synthesized from 1 µg total RNA with Superscript III (Invitrogen) using random hexamers following the manufacturer's instructions. All PCR experiments were performed with the annealing temperature at 57°C. Primer sequences used in this study are summarized in Table S1.

### Immunocytochemistry

Cultured cells were fixed with 4% PFA (paraformaldehyde)/PBS (phosphate-buffered saline) for 10 minutes at room temperature, washed with PBST (0.1% Triton X-100 in PBS), then pre-treated with blocking solution (3% BSA and 2% skim milk (DIFCO, USA) in PBST) at 4°C overnight. The cells were incubated with primary antibodies; anti-OCT4 (1∶50; Santa Cruz Biotechnology, USA), anti-SOX2 (1∶500; Abcam, UK) and anti-NANOG (1∶200; ReproCELL, Japan), anti-CDH1 (1∶200; TaKaRa, Japan), anti-SSEA4 (1∶500; Hybridoma Bank, USA), and anti-TRA-1-60 (1∶500; Millipore, USA) antibodies at 4°C overnight. They were then incubated with secondary antibodies; anti-rabbit IgG or anti-mouse IgG conjugated with Alexa 488 (1∶500; Molecular Probes, USA) in blocking buffer for 1 hour at room temperature. The cells were counterstained with DAPI (4,6-diamidino-2-phenylindole) and mounted with a SlowFade light antifade kit (Invitrogen).

### Tumor formation

A cell suspension of 5.0×10_5_ iESCs or iCSCs in 200 µl DMEM was subcutaneously injected into the inguinal region or transplanted into the kidney capsules of immunodeficient SCID mice (CLEA, Japan). Tumors were surgically dissected out 5–7 weeks after implantation, fixed with 4% paraformaldehyde in PBS and embedded in paraffin. Sections 5 µm in thickness were stained with hematoxylin and eosin.

### Microarray and data processing

For microarray analyses, 1 µg total RNA was labeled according to standard Affymetrix protocols and hybridized to the Affymetrix human genome U133 Plus 2.0 Array (samples of human origin). Raw data were normalized by the MAS 5.0 method using the bioconductor package on R program (http://www.r-project.org/). Heat maps of the gene expression profile for all genes in each cell line were visualized by MeV program (http://www.tm4.org/mev/). Hierarchical clusters were calculated by Pearson's correlation coefficient (r) and visualized by the pvclust package on R program. For scatter plot analyses, raw data were normalized by Robust Multichip Average (RMA). Scatter plots were visualized using the bioconductor package on R program.

## Results and Discussion

### Isolation of iESCs and iCSCs

**Figure 1:**
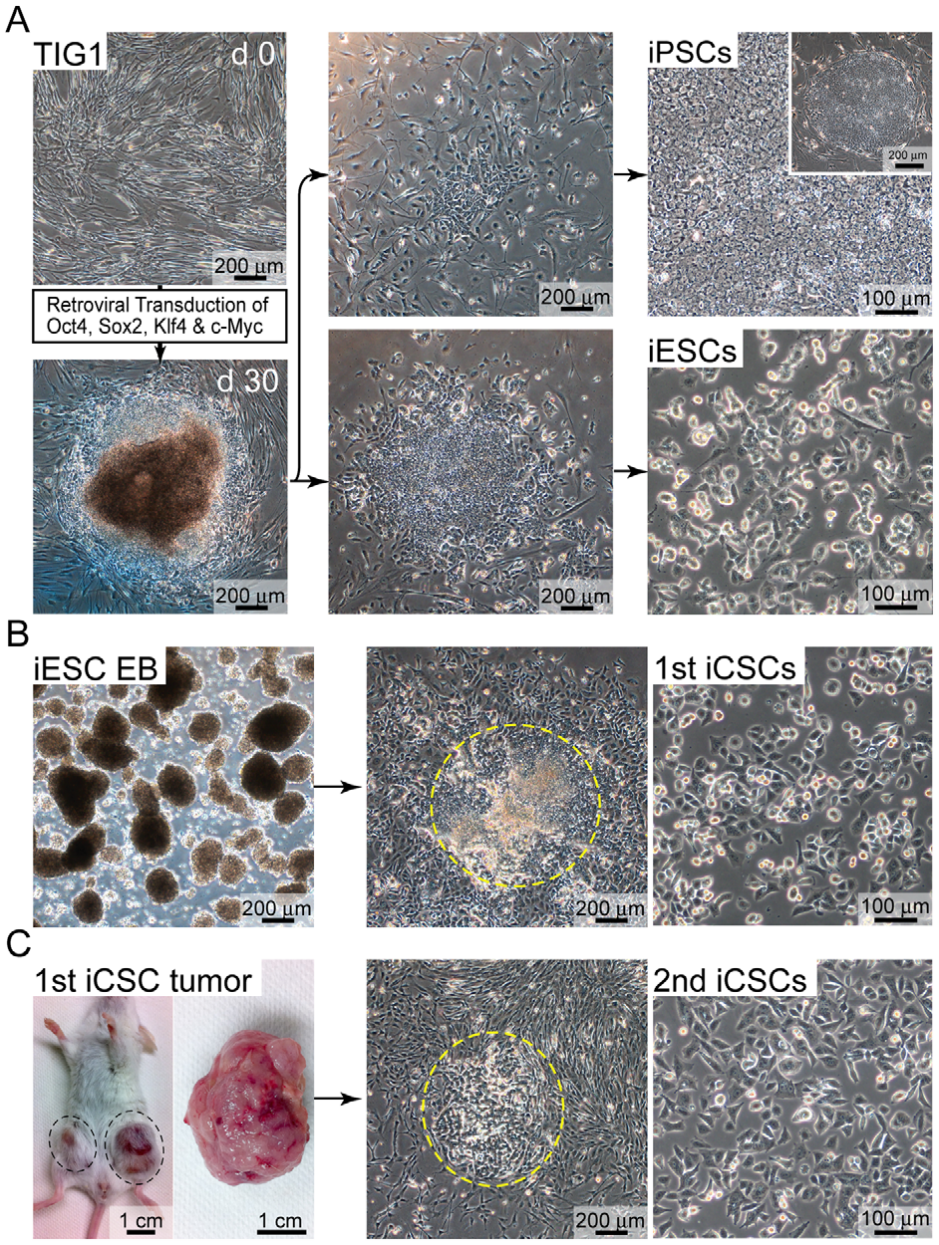
Generation of iPSC, induced epithelial stem cell (iESC), and induced cancer stem-like cell (iCSC) lines. (A) Generation of iPSCs and iESCs through direct reprogramming of primary fetal fibroblasts (TIG1). (B) Generation of the first (1st) iCSCs by picking a cell clump (yellow circle) through iESCs-derived embryoid bodies (iESC EB) formation. (C) Generation of the second (2nd) iCSCs by picking a cell clump (yellow circle) through serial transplantation of the 1st iCSCs.

**Figure 2:**
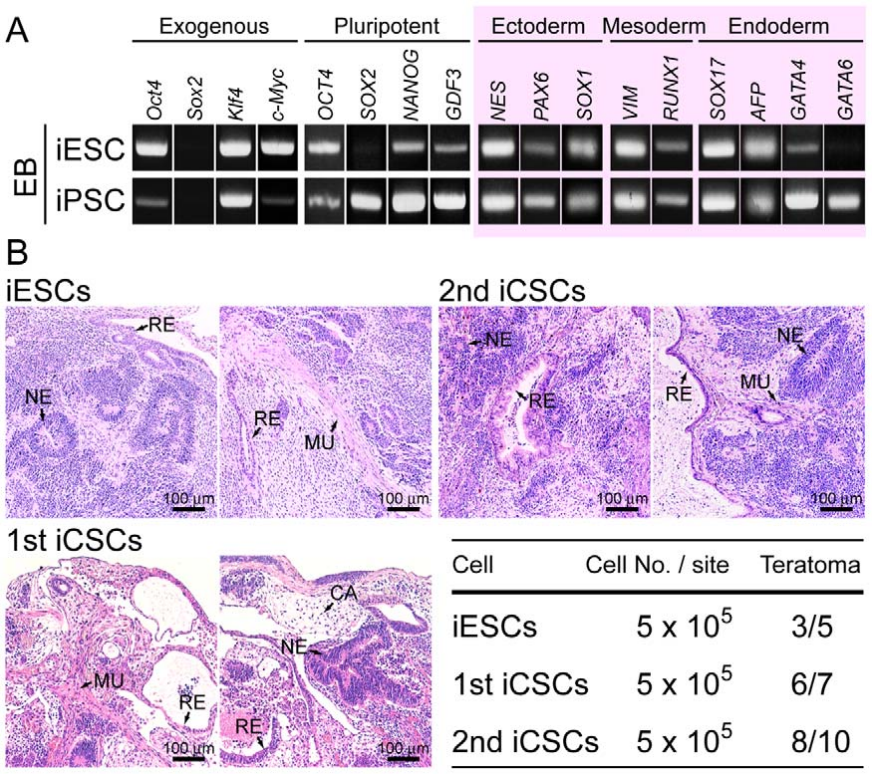
Pluripotent tumorigenicity of iESCs and iCSCs. (A) Expression of exogenous, pluripotent marker, and lineage marker genes in embryoid bodies (EBs) formed with iESCs and iPSCs by RT-PCR analyses. (B) Paraffin sections of teratomas, which were generated by iESCs, 1st iCSCs, and 2nd iCSCs implantation, were stained with hematoxylin and eosin. NE, neuroepithelium (ectoderm); CA, cartilage (ectoderm); MU, muscle (mesoderm); RE, respiratory epithelium (endoderm).

Human iPSCs were picked up as colonies about 30 days after retroviral transduction of Oct4, Sox2, Klf4 and c-Myc into the somatic fibroblasts TIG1, a cell line isolated from human fetal lung (Fig. 1A). The efficiency of iPSC generation was less than 1%, and the other populations of cells were differentially reprogrammed. Some of the differentially reprogrammed somatic cells showed the property of self-renewal formed colonies consisting of cells (iESCs) with epithelial cell morphology in MEF-conditioned iPSC medium (Fig. 1A). iESCs were maintained with epithelial cell morphology for around 10 passages, since were prone to differentiate (Fig. S1A). Therefore, to analyze the differentiation potential of iESCs, EB formation was induced by suspension culture (Fig. 1B). Successfully formed iESC EBs showing pluripotency as analyzed by RT-PCR (Fig. 2A) were attached on the bottom to culture dishes to isolate self-renewal stem cells. Consequently, the first (1st) iCSC lines isolated by picking cell clumps of expanded EBs in three independent experiments were stably maintained for more than 20 passages, or re-plated from frozen-stored cells (Fig. 1B). To verify the identity of the 1st iCSCs, the second (2nd) iCSCs were isolated from tumors generated by serial transplantation with injection of the 1st iCSCs into the inguinal regions of immunodeficient SCID mice in two independent experiments (Fig. 1C). The 2nd iCSCs resembling the 1st iCSCs were stably maintained with features of epithelial cell morphology and robust cell growth for more than 20 passages (Fig. 1C). Next, to explore the pluripotency of iESCs, 1st iCSCs, and 2nd CSCs, the cells were transplanted into kidney capsules or inguinal regions of SCID mice. Formation of teratomas containing ecotoderm, mesoderm, and endoderm derivatives was detected with hematoxylin and eosin staining at high frequency in all cell types (Fig. 2B), indicating that the three cell types were stem cells acquiring pluripotency, despite having epithelial cell morphology.

### Gene expression profile of iESCs and iCSCs

**Figure 3:**
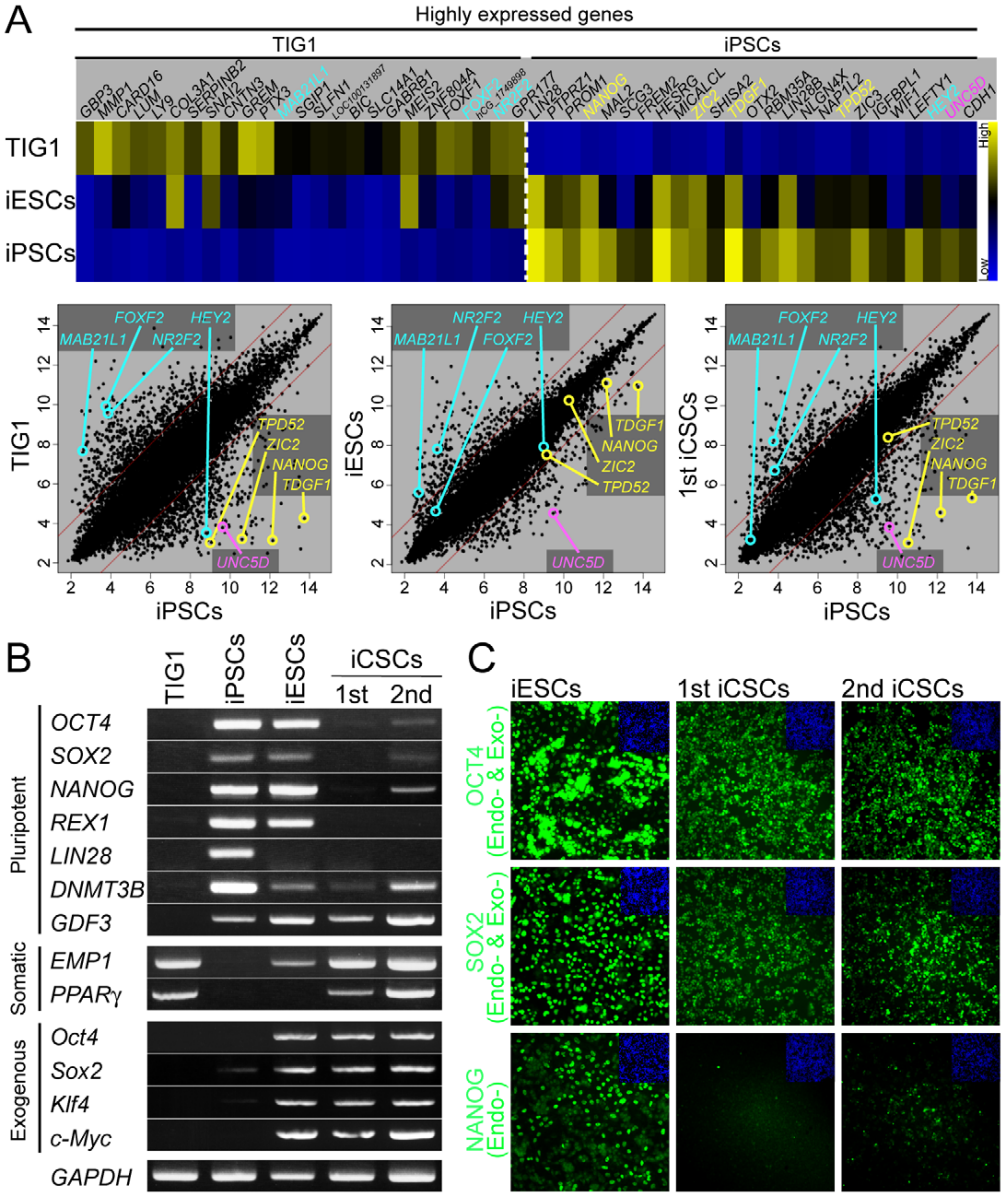
Global expression analysis of iESCs and iCSCs. (A) Gene expression microarray analysis among TIG1, iESCs, iCSCs, and iPSCs. Heat map (upper panel) shows differentially expressed genes of somatic and pluripotent genes. Scatter plots (lower panel) show comparison of global gene expression profiles. Yellow; genes in the Core module, Blue; genes in the PRC module, and Red; gene in the Myc module. (B) Expression analysis of pluripotent marker genes, somatic cell marker genes, and exogenous genes in TIG1, iPSCs, iESCs, 1st iCSCs, and 2nd iCSCs by RT-PCR analyses. (C) Expression of pluripotent marker proteins (Green) in iESCs, 1st iCSCs, and 2nd iCSCs by immunocytochemistry. Cell nuclei are counterstained blue with DAPI (Blue). Exo-, exogenous; Endo-, endogenous.

To examine the gene expression in iESCs and iCSCs, global gene expression profiles detected by gene expression microarray analyses were compared. Among TIG1, iESCs, 1st iCSCs, and iPSCs, iESCs and iCSCs closely resemble each other. Interestingly, iESCs and iCSCs were more similar to somatic cells, and TIG1 rather than human pluripotent cells (iPSCs and ES cells), even with the consistent high expression of the exogenous *Oct4*, *Sox2*, *Klf4*, and *c-Myc* (Fig. S2 and Fig. 3A and 3B). Consistent with this, data on heat map analyses demonstrated that iESCs maintained an intermediate state between somatic fibroblasts TIG1 and pluripotent iPSCs (Fig. 3A). In more detail, scatter plot analyses showed that, in iESCs, expression of somatic marker genes *MAB21L1* and *NR2F2* was high to iPSCs, whereas expression of pluripotent marker genes T*DGF1, NANOG, ZIC2,* and *TPD52* similar to iPSCs (Fig. 3A). Similar to iESCs, 1st iCSCs were characterized by expression of some somatic marker genes. RT-PCR analyses verified that the expression of endogenous pluripotent marker genes, *OCT4*, *SOX2*, *NANOG*, *REX1*, *LIN28,* was obviously low, while somatic marker genes, *EMP1*, *PPARγ*, *FOXF2*, and *NR2F2* were highly expressed in the 1st and 2nd iCSCs (Fig. 3A and 3B), indicating that epigenetic reprogramming went halfway to erasing the somatic memory and establishing a pluripotent transcription network. Low expression of NANOG was detected by immunocytochemistry in the 1st and 2nd iCSCs, while high expression of endogenous OCT4/exogenous Oct4 and endogenous SOX2/exogenous Sox2 was detected (Fig. 3C). Immunocytochemistry analyses demonstrated that pluripotent markers CDH1 and SSEA4 were weakly expressed in iESCs and iCSCs, while highly in iPSCs. Furthermore, expression of TRA-1-60 was detected in iPSCs, but not iESCs and iCSCs (Fig. S3). Collectively, iESCs and iCSCs retained a cellular state between TIG1 and iPSCs.

### Acquisision of pluripotency prior to establishment of iPSC identity

In the process of reprogramming somatic cells into iPSCs, cells progressively change their expression patterns and morphology. Thus, to compare gene expression profiles of different types of reprogramming cells, an integrated analysis system for expression profiles, in terms of the kinetics of modules, is required. Three ES cell modules, Core, PRC, and Myc (CPM), which are functionally separable, have been defined [9]. The Core module includes known factors in core regulatory circuitry, such as *NANOG, OCT4, SOX2, TCF3*, and *REX1*. The PRC module includes gene generally repressed in ES cells, including *HOX* cluster genes. The Myc module is composed of genes that are common targets of seven factors, MYC, MAX, NMYC, DMAP1, E2F1, E2F4, and ZFX. In addition, the ES cell-like module was defined to distinguish among ES cells, adult tissue stem cells, and human cancers [13]. Here, we demonstrate data with CPM modules, since analysis with data of the ES cell-like module was comparable to data of the Core module of CPM modules, which included pluripotent marker genes.

**Figure 4:**
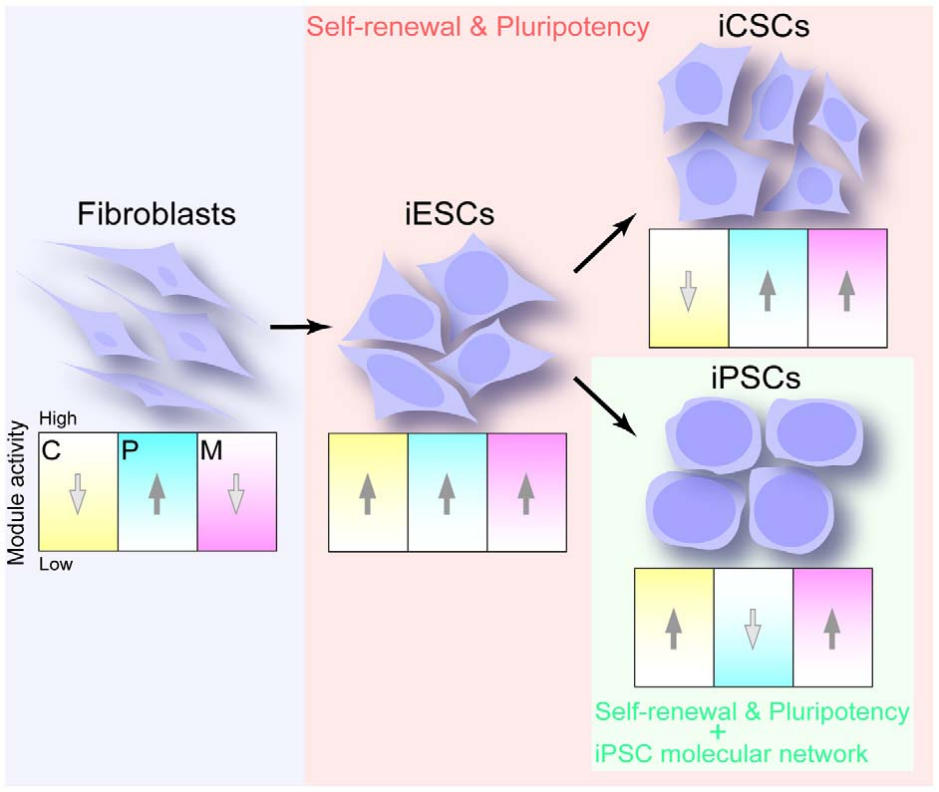
Activity of CPM modules in human somatic cells and pluripotent stem cells. Properties of self-renewal and pluripotency are linked with transient or continuous up-regulation of the Core and Myc modules, but not PRC module. C, Core module; P, PRC module; M, Myc module.

To reprogram TIG1 into iESCs, up-regulation of the Core and Myc modules was required (Fig. 4 and Fig. S4). iESCs and iCSCs were characterized by the high activity of the PRC and Myc modules, while the Core module was high in iESCs and low in iCSCs. It was crucial to impose a barrier for erasing somatic memory that the PRC module retained high activity in all TIG1, iESCs, and iCSCs as shown by *FOXF2* and *NR2F2* (Fig. 3A). To be fully reprogrammed into iPSCs from TIG1, induction of the down-regulation of the PRC module was necessary (Fig. 4 and Fig. S4), indicating that completion of the establishment of the iPSC-transcriptional network is associated with down-regulation of the PRC module.

Stable cell lines, TIG1, iCSC, and iPSC, were characterized by the reciprocal activity of the Core and PRC modules, while unstable iESCs showed simultaneous up-regulation of the two modules, suggesting that genes categorized into the two modules functioned competitively in reprogramming. Notably, acquisition of self-renewal and pluripotency was linked with up-regulation of the Core and Myc, but not PRC, modules (Fig. 4). These data indicated that the property of self-renewal and pluripotency could be conferred on somatic nuclei prior to full reprogramming into iPSCs. The finding that a certain cell population of iESCs was reprogrammed into iPSCs spontaneously supported this concept (Fig. S1B). The criteria of self-renewal and pluripotency are widely used for defining human iPSCs. However, the property of self-renewal and pluripotency can be conferred on a variety of cell types through reprogramming more than we expected, and are necessary, but not sufficient, for defining iPSC identity.

To reprogram somatic cells to iCSCs, up-regulation of the Myc module is a key event. Promoter regions bound by MYC are linked with histone H3 lysin4 trimethylation (H3K3me3), which is positively correlated with the formation of open chromatin, and gene activation as an epigenetic signature [9]. Furthermore, MYC interacts with histone acetyltransferases, which are associated with transcription activation complexes [14]. The Myc module facilitates cell metabolism by activating general genes. Genes in the Core module are also up-regulated through somatic reprogramming to iCSCs. *OCT4*, *SOX2*, and *NANOG*, which are key Core module players, repress developmentally important homeodomain proteins through co-occupation of their target genes, while promote self-renewal and pluripotency by positive regulation of genes encoding components of key signaling pathways [15]. Taking these into consideration, the Core and Myc modules play a role in conferring the features of self-renewal and pluripotency on somatic nuclei, whereas continuously high activity of the PRC module in TIG1, iESCs, and iCSCs impedes the resetting of the somatic memory of some developmentally important homeodomain proteins (Fig. 4). The polycomb repressive complexes, especially polycomb repressive complex 2 containing Ezh2, Eed, and Suz12, are crucial repressors of genes in association with H3K27me3 [16]. In order to reset the somatic memory of genes with a homeodomain in iESCs and iCSCs and over-write the ES cell-like epigenetic signature in the reprogrammed nuclei of iESCs, the activity of the PRC module should be reduced continuously or transiently. Relatively reduced activity of the PRC module may be a key event for reprogramming somatic cells to iPSCs. It is speculated that lack of H3K27me3 plays important roles in promoting reprogramming from pre-iPSCs to iPSCs in the late phase of reprogramming [17]. The fate of somatic cells, whether they are reprogrammed to iCSCs or iPSCs, could be determined by activity of the PRC module, after acquiring the capability of self-renewal and pluripotency (Fig. 4).

## Supporting Information

Figure S1 Spontaneous differentiation of iESCs and conversion into iPSCs. (A) Differentiation of iESCs after long-term culture. (B) Conventional iPSCs (right panel) were generated from a small colony (yellow circle in left panel) appearing in iESC culture at high cell density.
(TIF)

Figure S2 Global gene expression analysis of iESCs and iCSCs. Comparative analysis of global gene expression profile in iPSC, human embryonic stem (ES) cell, iESC, 1st iCSC, and TIG1 lines by gene expression microarray assay.
(TIF)

Figure S3 Expression of pluripotent marker proteins in iESCs and iCSCs. Expression of marker cell surface proteins, CDH1, SSEA4, and TRA-1-60 was detected as green fluorescence, while cell nuclei were as blue with DAPI.
(TIF)

Figure S4 Average gene expression values (log2) of CPM modules in in somatic cells and pluripotent stem cells.
(TIF)

Table S1 Primers for RT-PCR analyses.
(DOC)
