# Supplementary figures and images for: Self-Renewal and Pluripotency Acquired through Somatic Reprogramming to Human Cancer Stem Cells
Source: PLoS One. 2012 Nov 8;7(11):e48699. doi: 10.1371/journal.pone.0048699 (PMC3493587; doi:10.1371/journal.pone.0048699)

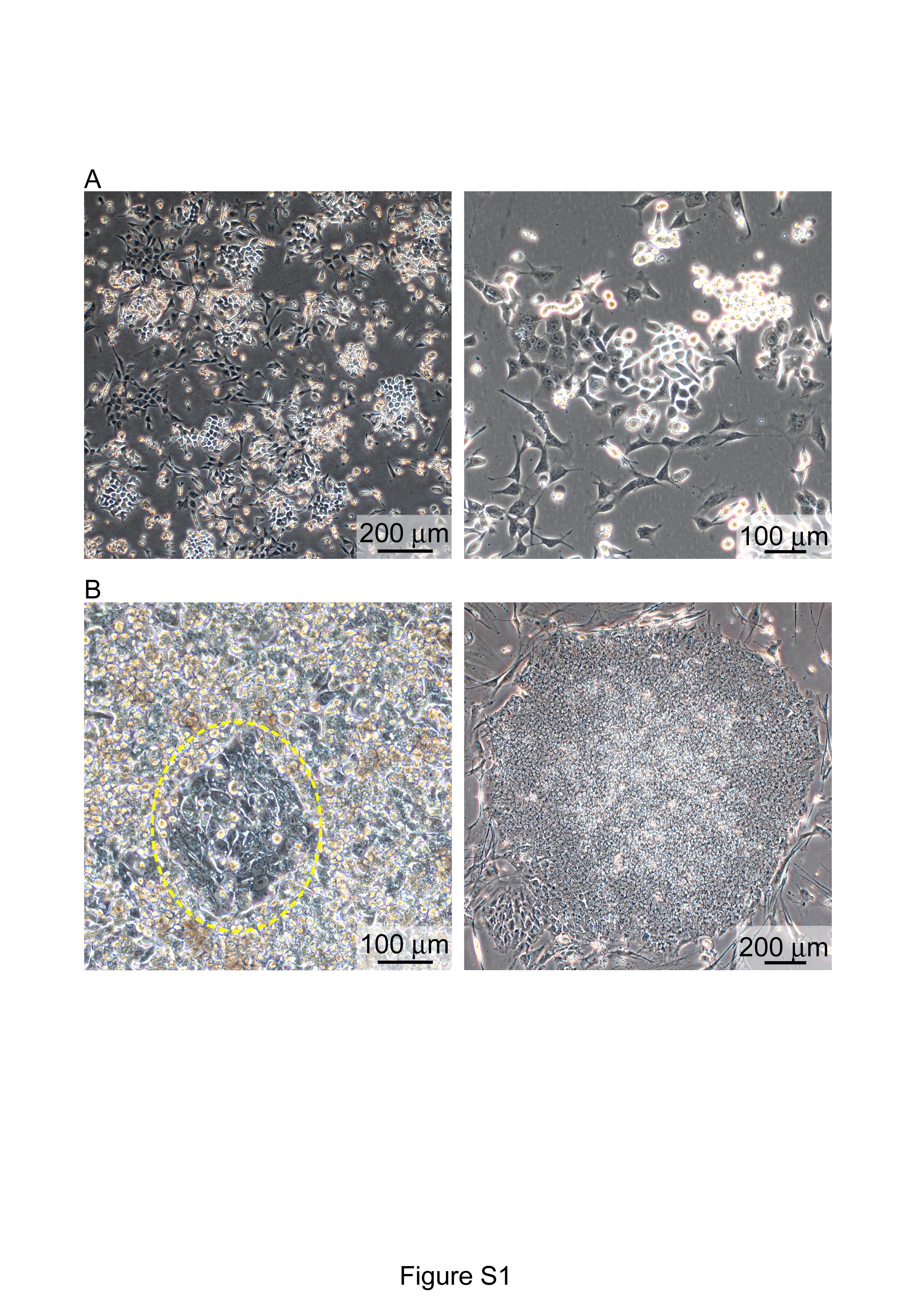

Supplement: Figure S1 — Spontaneous differentiation of iESCs and conversion into iPSCs. (A) Differentiation of iESCs after long-term culture. (B) Conventional iPSCs (right panel) were generated from a small colony (yellow circle in left panel) appearing in iESC culture at high cell density. (TIF) [file pone.0048699.s001.tif]

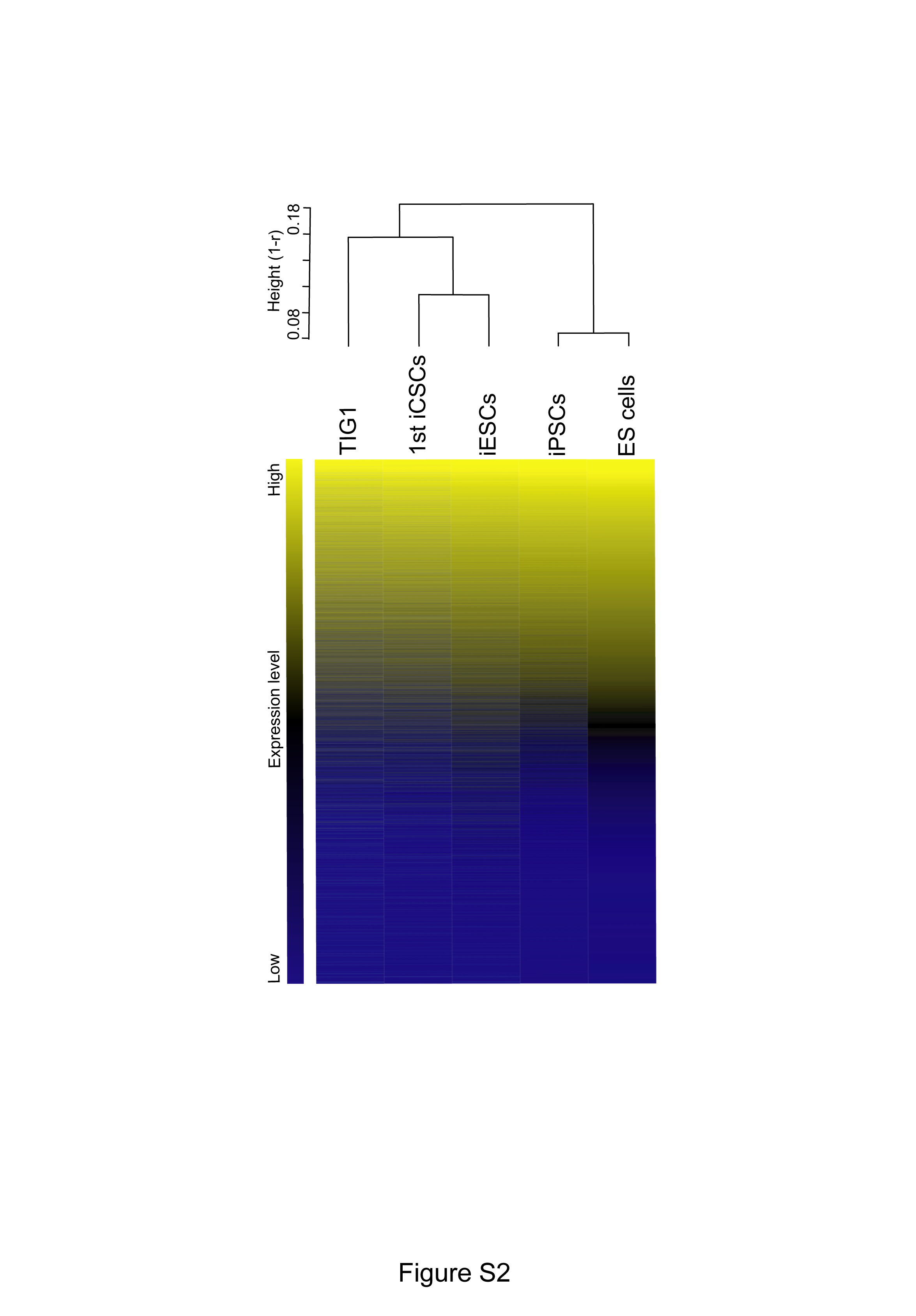

Supplement: Figure S2 — Global gene expression analysis of iESCs and iCSCs. Comparative analysis of global gene expression profile in iPSC, human embryonic stem (ES) cell, iESC, 1st iCSC, and TIG1 lines by gene expression microarray assay. (TIF) [file pone.0048699.s002.tif]

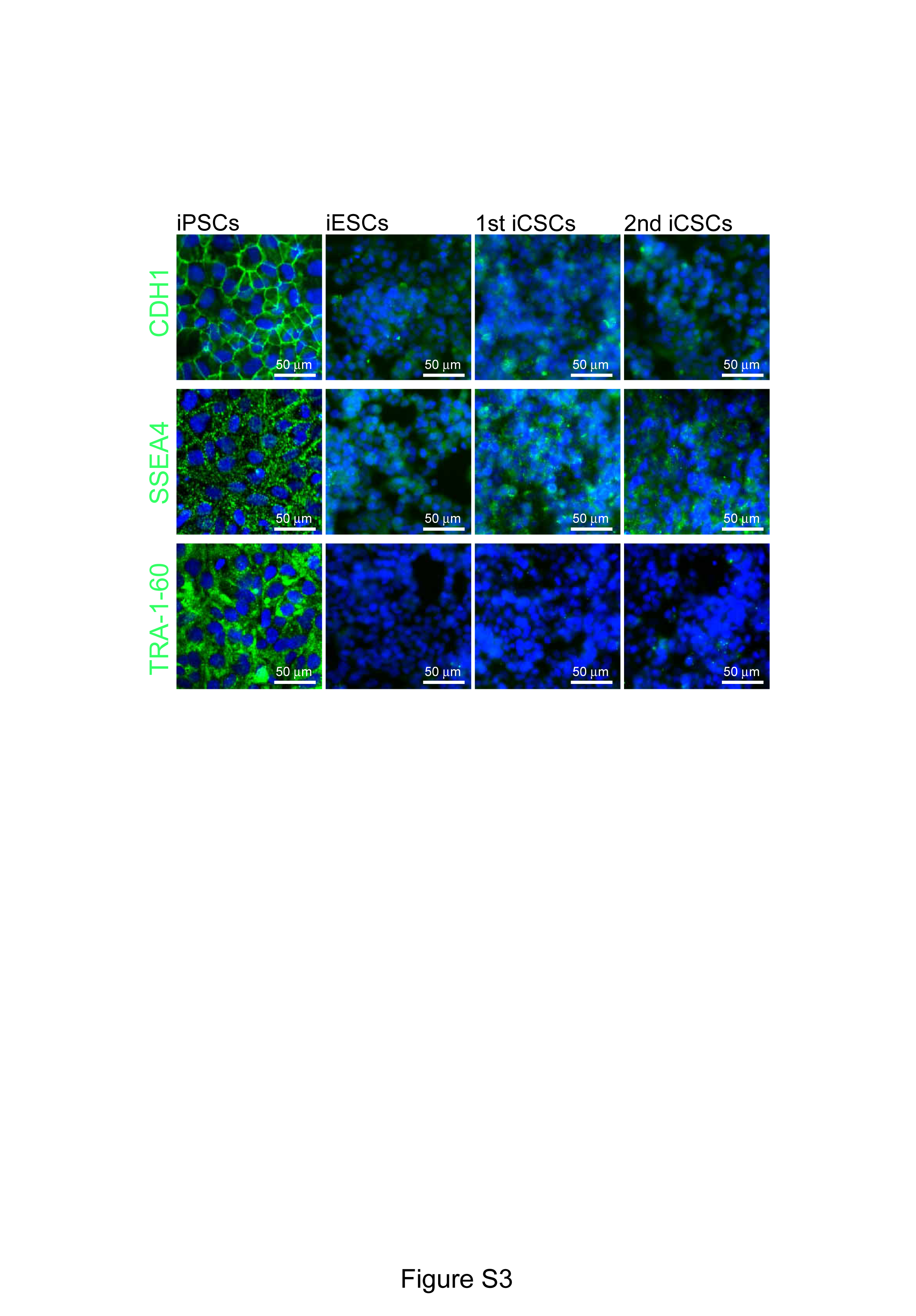

Supplement: Figure S3 — Expression of pluripotent marker proteins in iESCs and iCSCs. Expression of marker cell surface proteins, CDH1, SSEA4, and TRA-1-60 was detected as green fluorescence, while cell nuclei were as blue with DAPI. (TIF) [file pone.0048699.s003.tif]

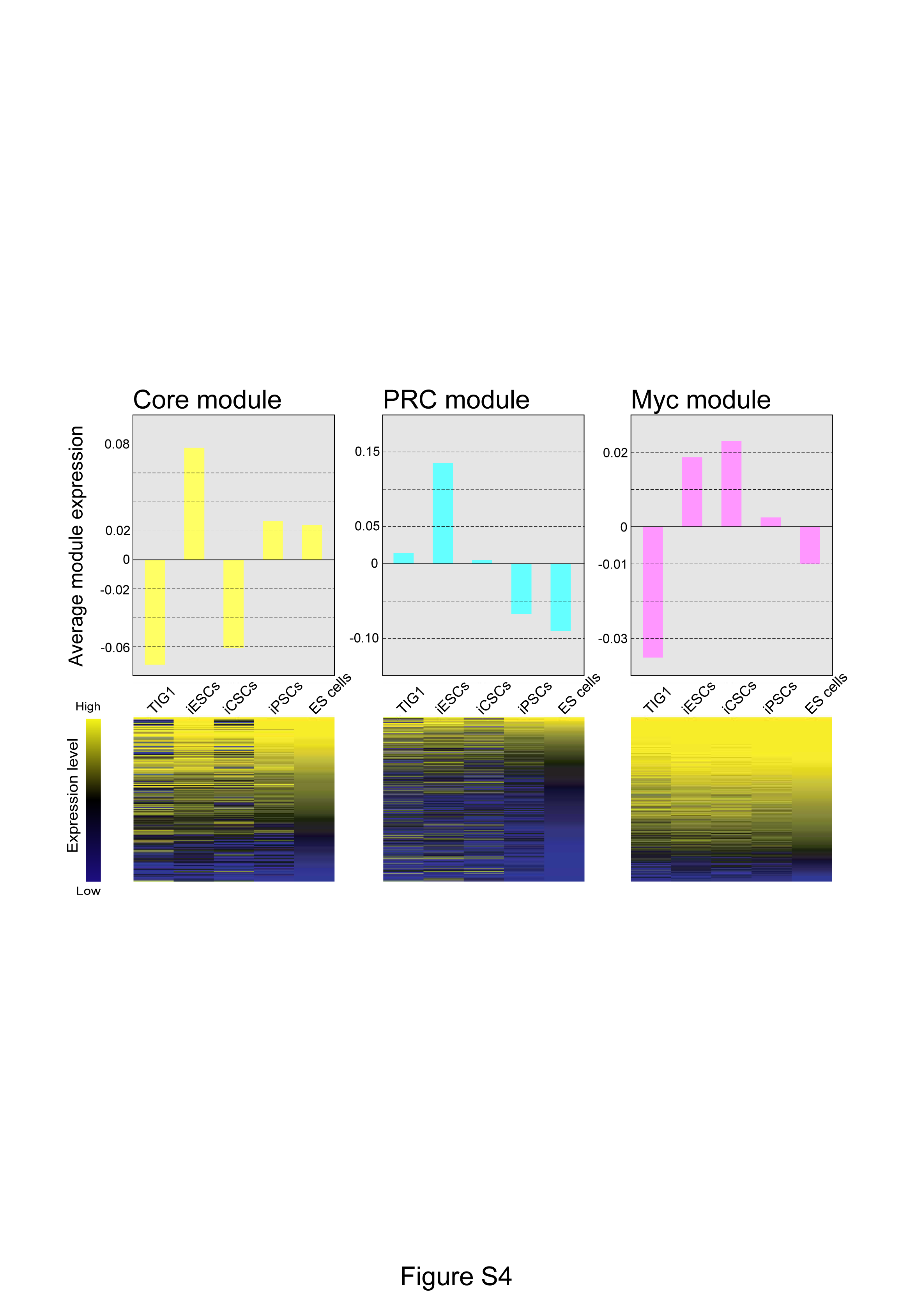

Supplement: Figure S4 — Average gene expression values (log2) of CPM modules in in somatic cells and pluripotent stem cells. (TIF) [file pone.0048699.s004.tif]
