# Supplementary material for: Self-Renewal and Pluripotency Acquired through Somatic Reprogramming to Human Cancer Stem Cells
Source: PLoS One. 2012 Nov 8;7(11):e48699. doi: 10.1371/journal.pone.0048699 (PMC3493587; doi:10.1371/journal.pone.0048699)
Supplement: Table S1 — Primers for RT-PCR analyses. (DOC) [file pone.0048699.s005.doc]

| gene | Forward (5’-3’) | Reverse (5’-3’) |
| --- | --- | --- |
| *Oct4 (transgene)* | CCCATGGTGGTGGTACGGGAATTC | AGTTGCTTTCCACTCGTGCT |
| *Sox2 (transgene)* | CCCATGGTGGTGGTACGGGAATTC | TCTCGGTCTCGGACAAAAGT |
| *Klf4 (transgene)* | CCCATGGTGGTGGTACGGGAATTC | GTCGTTGAACTCCTCGGTCT |
| *c-Myc (transgene)* | CTCCTGGCAAAAGGTCAGAG | GACATGGCCTGCCCGGTTATTATT |
| *OCT4* | GCACTGTACTCCTCGGTCCCTTTCCC | CTTCCCTCCAACCAGTTGCCCCAAAC |
| *SOX2* | GGGAAATGGGAGGGGTGCAAAAGAGG | TTGCGTGAGTGTGGATGGGATTGGTG |
| *NANOG* | CAGCCCTGATTCTTCCACCAGTCCC | TGGAAGGTTCCCAGTCGGGTTCACC |
| *GDF3* | CTTATGCTACGTAAAGGAGCGGG | GTGCCAACCCAGGTCCCGGAAGTT |
| *NES* | TCCAGGAACGGAAAATCAAG | GCCTCCTCATCCCCTACTTC |
| *PAX6* | GTCCATCTTTGCTTGGGAAA | TAGCCAGGTTGCGAAGAACT |
| *SOX1* | CACAACTCGGAGATCAGCAA | GGTACTTGTAATCCGGGTGC |
| *RUNX1* | CCCTAGGGGATGTTCCAGAT | TGAAGCTTTTCCCTCTTCCA |
| *VIM* | GTCCCTGGAACGCCAGATGCG | CCTGCAGGCGGCCAATAGTGT |
| *GATA4* | CTAGACCGTGGGTTTTGCAT | TGGGTTAAGTGCCCCTGTAG |
| *SOX17* | CGCTTTCATGGTGTGGGCTAAGGACG | TAGTTGGGGTGGTCCTGCATGTGCTG |
| *AFP* | ACTGAATCCAGAACACTGCATAG | GCTTCTTGAACAAACTGGGCAAA |
| *GATA6* | AGGGCTCGGTGAGTCCAAT | CGCTGCTGGTGAATAAAAAGGA |
| *REX1* | CAGATCCTAAACAGCTCGCAGAAT | GCGTACGCAAATTAAAGTCCAGA |
| *LIN28* | TGCGGGCATCTGTAAGTGG | GGAACCCTTCCATGTGCAG |
| *DNMT3B* | TGCTGCTCACAGGGCCCGATACTTC | TCCTTTCGAGCTTCAGTGCACCACAAAAC |
| *EMP1* | GCTGTCCCTCATGGAGACCT | AAGTGGGATAGGCAGGGTCC |
| *PPARγ* | CGTGGCCGCAGATTTGAAAG | CCATGAGGGAGTTGGAAGGC |
| *GAPDH* | CTTCTTTTGCGTCGCCAGCCGAG | CAGCCTTGACGGTGCCATGGAA |

Table S1
